# Mapping Insight Dimensions and Symptom Dynamics in Schizophrenia: A Data-Driven Network Approach: Cartographie des dimensions d’insight et de la dynamique symptomatique dans la schizophrénie: une approche par réseau fondée sur les données

**DOI:** 10.1177/07067437251329074

**Published:** 2025-03-21

**Authors:** Jesse Rae, Katie M. Lavigne, Geneviève Sauvé, Martin Lepage, Delphine Raucher-Chéné

**Affiliations:** 1 548619 Douglas Research Centre, Montreal, QC, Canada; 2 Département de Psychologie, Université de Montréal, Montreal, QC, Canada; 3 Department of Psychiatry, 5620 McGill University, Montreal, QC, Canada; 4 Département d’Éducation et Pédagogie, Université du Québec à Montréal, Montreal, QC, Canada

**Keywords:** clinical insight, awareness of illness, symptom attribution, need for treatment, depression, anxiety

## Abstract

**Objectives:**

Patients with *schizophrenia spectrum disorders* (SSD) present with cognitive, behavioral, and emotional difficulties. Affected individuals often exhibit poor insight into aspects of their illness, such as awareness of the illness itself or the need for treatment, which can hinder treatment adherence and complicate clinical outcomes. This study aimed to investigate the relationships between clinical symptoms and dimensions of insight in SSD using a network approach, which captures direct and indirect relationships among variables. We hypothesized that illness awareness would correlate negatively with positive symptoms and positively with depressive symptoms, and that positive symptoms would have the strongest influence on the network.

**Methods:**

Data were collected from 142 individuals diagnosed with SSD. Insight was measured using the Birchwood Insight Scale (IS) across three dimensions: illness awareness, symptom re-labelling, and awareness of the need for treatment. Symptoms were evaluated using the Scale for the Assessment of Positive Symptoms, the Scale for the Assessment of Negative Symptoms, the Calgary Depression Scale and the Hamilton Anxiety Scale. Network analysis was employed to explore interconnections (edges) between variables (nodes) and identify influential variables through centrality measures (strength, betweenness, closeness).

**Results:**

A significant positive connection was found between illness awareness and depressive symptoms. Anxiety and depressive symptoms were identified as the most central and influential variables within the network. Treatment awareness showed greater centrality than illness awareness, indicating this dimension's potential importance in influencing symptom dynamics in a clinical profile.

**Conclusions:**

Analyzing a more extensive network that includes treatment adherence and cognitive domains affected in SSD could enhance and validate the understanding of the cascading effects of symptoms and insight dimensions, allowing for more tailored treatments.

## Plain Language Summary Title

Interconnections between levels of awareness and clinical symptoms in schizophrenia and related disorders

Plain Language Summary

Schizophrenia is a complex mental illness that affect thinking, emotions, and behaviors. Many individuals with schizophrenia struggle to recognize that they are ill or their need for treatment, which complicates adherence to treatment plans. This study aimed to explore how different symptom domains of schizophrenia are connected to aspects of a person's insight into their illness.

Data was collected from 142 people diagnosed with schizophrenia spectrum disorders. Insight was measured using the Birchwood Insight Scale, which assesses three key dimensions: how aware people are of their illness, how they label or recognize their symptoms, and how aware they are of the need for treatment. Symptom domains in schizophrenia, including positive symptoms (e.g., delusions, hallucinations), negative symptoms (e.g., affect flattening, alogia), depression, and anxiety, were assessed. A network analysis analyzed the relationships between insight dimensions, symptom domains, and subdomains. In this approach, each variable is represented as a “node” in a network, and its connections are shown as “edges.” The strength of these connections can reveal which variables are most important in influencing others.

Greater awareness of one's illness was linked to greater depressive symptoms. Anxiety and depression emerged as the most influential symptoms in the network, meaning they had strong connections with other symptoms. Awareness of the need for treatment was the most influential insight dimension within the network, suggesting that understanding the need for treatment may play an essential role in managing symptoms. A larger sample, including other clinical outcome variables within the network, such as treatment adherence and functioning, could provide a better understanding of how symptoms interact and lead to various clinical outcomes.

## Abstract (fr)

**Objectif:**

Les personnes atteintes de *troubles du spectre schizophrénique* (TSS) éprouvent des difficultés cognitives, comportementales et émotionnelles. Elles affichent souvent un mauvais insight dans des aspects de leur maladie, comme la conscience de la maladie elle-même ou du besoin de traitement, ce qui peut entraver l’observance thérapeutique et compliquer les résultats cliniques. Cette étude avait pour but d’explorer les relations entre les symptômes cliniques et les dimensions d’insight dans les TSS à l’aide d’une analyse en réseau, qui capture les relations directes et indirectes entre les variables. Nous avons postulé que la conscience de la maladie serait corrélée négativement avec les symptômes positifs et positivement avec les symptômes dépressifs et que les symptômes positifs exerceraient la plus forte influence sur le réseau.

***Méthodologie*:**

Des données ont été recueillies auprès de 142 personnes ayant un TSS. L’insight a été mesuré avec l’échelle de Birchwood dans trois dimensions : conscience de la maladie, conscience des symptômes et conscience du besoin de traitement. Les symptômes ont été évalués à l’aide de l’échelle des symptômes positifs, l’échelle des symptômes négatifs, l’échelle de dépression de Calgary et l’échelle d’anxiété de Hamilton. Une analyse en réseau a été utilisée pour explorer les interconnexions (arêtes) entre les variables (nœuds) et déterminer les variables influentes au moyen de mesures de centralité (degré, intermédiarité, proximité).

Une connexion positive significative a été observée entre la conscience de la maladie et les symptômes dépressifs. L’anxiété et les symptômes dépressifs ont été définis comme variables les plus centrales et importantes du réseau. La conscience du besoin de traitement montrait une plus grande centralité que la conscience de la maladie, ce qui indique l’importance potentielle de cette dimension pour influer sur la dynamique des symptômes dans un profil clinique.

L’analyse d’un réseau plus large incluant l’adhésion au traitement et les domaines cognitifs d’intérêt dans les TSS pourrait hausser et valider la compréhension des effets en chaîne des symptômes et des dimensions d’insight et permettre ainsi de mieux cibler les traitements.

## Introduction

*Schizophrenia* is a severe mental illness characterized by various cognitive, behavioral, and emotional difficulties. Variations in clinical insight, that is, the awareness or understanding of one's condition, in individuals with schizophrenia spectrum disorders (SSD) have been recognized as a significant aspect of the illness and have since been linked to adverse clinical outcomes, such as a failure to recognize the need for treatment, leading to poor or nonadherence.^1–3^ Better insight in this population has also been associated with less global psychopathology and better long-term functioning.^3^ While it is generally assumed that increased awareness of the severity of one's mental disorder is beneficial to treatment adherence, this process can be distressing for patients, partly due to perceived discrimination and devaluation and internalized stigma.^4–8^ Good insight has been linked to depressive symptoms during and post-psychosis.^9–11^

Emerging research on the association between clinical insight and symptom domains has revealed negative associations between insight and positive symptoms, particularly with delusions.^3,12–16^ Delusions, by their nature, often reflect a lack of awareness across multiple dimensions of insight. Patients with strong delusions may hold mistaken beliefs stemming from misinterpretations of their perceptions and maintain these beliefs even when faced with contradictory evidence, leading to reduced insight into their illness and difficulty in accurately perceiving their symptoms.^14^ Negative symptoms, which refer to reduced “normal” behaviors related to motivation and interest (e.g., avolition, anhedonia, asociality) or emotional expression (e.g., blunted affect, alogia), have also been negatively associated with clinical insight in several studies.^1,9,12,14,17,18^ One potential explanation for this relationship is that patients exhibiting negative symptoms, such as apathy and social withdrawal, often struggle to maintain strong therapeutic relationships and adhere to treatment plans which may explain their deficits in insight even after improvements in positive symptoms.^19^ The relationship between insight and affective symptoms (depression, anxiety) generally aligns with the insight paradox previously mentioned, showing that increased insight is associated with more depressive and anxiety symptoms.^9,10,20^

It is now generally agreed that insight is not a simple, all-or-none phenomenon based solely on illness acceptance but rather a complex, multidimensional construct.^21–25^ However, many studies still represent insight as unidimensional using a global rating or total insight score that fails to differentiate between specific dimensions, such as awareness of one's illness, recognizing (or relabelling) one's symptoms as pathological, and awareness of one's need for treatment.^26^ Illness awareness focuses on recognizing and acknowledging the presence of a psychiatric disorder. Relabelling of symptoms involves understanding that specific symptoms or signs are pathological and associated with the condition. Awareness of the need for treatment involves the recognition of the necessity of professional intervention based on presenting difficulties.^26^ These dimensions have been shown to vary independently; thus, relying on a global insight rating can miss these nuanced distinctions and inaccurately reflect patients’ experiences.^27^ Identifying how different dimensions of insight influence symptoms, and vice versa, can help clinicians tailor interventions to address each patient's unique needs better.

Traditional statistical approaches primarily focus on examining individual associations between symptoms and insight dimensions, often overlooking the interactions and dependencies that may exist within the network. Network theory of mental disorders conceptualizes psychopathology as a system of interconnected symptoms, suggesting that symptoms interact to cause, exacerbate, or associate with the expression of other symptoms.^28,29^ Network analysis, a data-driven statistical technique, constructs symptom networks, revealing any direct and indirect relationships between variables. For instance, a network analysis could show that auditory hallucinations may be linked to anxiety, which is linked to social withdrawal and depressive symptoms. Instead of analyzing each symptom in isolation, this approach recognizes that no single symptom can be examined without considering the other symptoms’ influence.^30–32^

This method uses centrality indices to determine the type and degree of influence of each variable in the network: node strength, closeness, and betweenness. In symptom networks, variables with high centrality may be critical areas for intervention, as they likely influence other symptoms in the network significantly. Thus, addressing these variables in treatment may lead to broader improvements across multiple areas of functioning.^33^ One recent systematic review of schizophrenia symptom networks reported that positive symptoms, specifically delusions, along with affective symptoms, are the most central and likely to influence other symptoms in SSD.^34^ Including insight dimensions in such a network could enhance our understanding of the clinical profile in SSD by revealing how these dimensions connect to and influence symptoms. This more comprehensive view could, in turn, allow for the development of more tailored treatments. Symptoms or insight dimensions with high centrality within the network could be prioritized in intervention, as addressing them could lead to broader improvements across the entire symptom network.

The current study aims to better understand the interrelations between clinical insight dimensions and symptom domains and subdomains in SSD using a network approach. Our first hypothesis was that *illness awareness* would be negatively associated with positive symptoms and positively associated with depressive symptoms. We also hypothesized that positive symptoms would exhibit the greatest strength within the network. Network analysis effectively addresses these points as it goes beyond examining isolated relations, revealing which variables most strongly influence the symptom network, highlighting key areas for intervention.

## Material and Methods

### Patients

Recruitment was conducted at the Douglas Mental Health University Institute and affiliated external resources. Initially, 165 participants were recruited, but due to missing data, the final sample included 142 participants aged 18–50, meeting diagnostic criteria for SSD (schizophrenia, schizoaffective disorder, bipolar disorder with psychotic features, delusional disorder), confirmed by the Structured Clinical Interview for DSM-IV. All participants were in an enduring phase of the disorder, defined as receiving pharmacological treatment for psychosis for at least 3 years. Participants spoke English or French and had otherwise good physical health. Exclusion criteria were a low IQ score (> two standard deviations below the group mean), a history of a medical or neurological condition, a family history of hereditary neurological disorder (e.g., Alzheimer's disease), or current substance dependence.

A trained research assistant or coordinator conducted a semi-structured interview to identify the age of illness onset as well as the duration of illness. Clinical data, including the number and duration of hospitalizations, current medications, and corresponding dosages, were confirmed by medical chart consultation. The study procedure conformed to the latest version of the Declaration of Helsinki. The research protocol was approved by the Douglas Institute's Research Ethics Committee. The nature of the study procedure was explained to all participants, followed by their written informed consent. Data collection occurred from November 2011 to June 2015.

### Clinical Measures

***Insight.*** The Birchwood Insight Scale (IS) is a self-report questionnaire that comprises eight items grouped into three subscales: (a) awareness of having a mental illness; (b) awareness of the need for treatment; (c) re-labelling of symptoms (recognizing one's symptoms as pathological). Each item is rated on a 4-point Likert scale, with higher scores indicating better insight. This measure demonstrated satisfactory 1-week test-retest reliability.^35^ ***Positive and negative symptoms.*** The Scale for the Assessment of Positive Symptoms (SAPS) and the Scale for the Assessment of Negative Symptoms (SANS) are clinician-rated scales to quantify positive and negative symptoms, respectively.^36,37^ The SAPS comprise 34 items, grouped into four sub-domains: hallucinations, delusions, bizarre behavior, and positive formal thought disorder. Each item is rated on a 6-point Likert scale for both scales, with higher scores indicating more severe. The SANS comprises 25 items, grouped into five sub-domains: affective blunting, alogia, avolition/apathy, anhedonia/asociality, and attention. The attention domain was excluded from the current study due to recent factor analytic studies showing that it does not cluster with the negative symptom factors.^38^ These two scales demonstrated strong interrater reliability and internal consistency for composite scores.^39^

***Affective symptoms.*** The Calgary Depression Scale (CDS) is a 9-item clinician-rated scale to evaluate depressive symptoms in individuals with schizophrenia or related disorders by distinguishing depressive symptoms from negative and extrapyramidal symptoms.^40^ Scores range from 0 to 27, with higher scores indicating more severe depressive symptomatology. This scale has shown strong interrater reliability and acceptable internal consistency.^41^ The Hamilton Anxiety Scale (HAM-A) is a 14-item clinician-rated scale to evaluate psychological and somatic anxiety symptoms.^42^ Each item is scored on a 4-point Likert scale; thus, scores can range from 0 to 56, with a higher score indicating more severe anxiety symptoms. This scale has shown strong test-retest reliability, and inter-rater reliability.^43^

### Statistical Analysis

Descriptive statistics and zero-order correlations were examined among the variables of interest. Subsequently, a network analysis was conducted using the R programming language (RR Core Team, 2013). The network structure was estimated for 7 variables: symptom domains’ (SAPS, SANS), Birchwood IS dimensions, and the CDS and HAS global scores. An exploratory analysis was also conducted, including 13 variables: subdomains of positive symptoms (hallucinations, delusions, bizarre behavior, thought disorder), negative symptoms (alogia, avolition, blunted affect, anhedonia), Birchwood IS dimensions, and the CDS and HAS global scores. Details of the network analyses are provided in the supplement. Node strength, closeness, and betweenness were assessed, which provide specifics about the structure and dynamics of the network. Node strength is the sum of all its partial correlations, quantifying the overall strength of a node's connections with other nodes. Node closeness reflects the closeness or distance from one node to others, highlighting its potential reach and efficiency in transmitting information. Node betweenness represents the number of times a particular node lies on the shortest path between two other nodes, indicating its potential role in facilitating or mediating communication within the network.^30^

The bootnet package was used to evaluate network accuracy and stability, following a well-established estimation routine involving (a) bootstrapping 95% confidence intervals of the edge weights, (b) assessing the stability of centrality indices through subset bootstrap, and (c) testing the significance of differences in edge weights against a null model.^30^

## Results

Table 1 presents the key demographic and clinical features of the participants included in the study (*N* = 141). Within the sample, 100 (70.9%) participants were diagnosed with schizophrenia, 30 (21.2%) with schizoaffective disorder, 10 (7.1%) with bipolar disorder with psychotic features, and 1 (0.07%) with delusional disorder.

**Table 1.** Demographic and Clinical Characteristics.

	*M* (SD)	Range
Male, *n* (%)	104 (73.8)	
Age (years)	35.7 (7.93)	21–50
Education (years)	11.32 (2.54)	4–22
IQ	94.6 (14.2)	66–134
Age of onset (years)	22.18 (6.63)	8–44
Duration of illness (years)	13.51 (7.9)	3–37
# of hospitalizations [*n* = 132]	4.81 (3.86)	0–20
**Symptom variables**		
Birchwood Insight Scale		
*Re-labelling of symptoms*	2.92 (1.06)	
*Illness awareness*	2.02 (1.33)	
*Awareness of the need for treatment*	3.43 (0.9)	
SAPS	18.71 (16.88)	
*Hallucinations*	2.13 (1.86)	
*Delusions*	2.11 (1.69)	
*Behavior*	1.10 (1.17)	
*Thought disorder*	1.35 (1.37)	
SANS	23.04 (10.08)	
*Affect flattening*	2.33 (1.2)	
*Alogia*	1.50 (1.19)	
*Avolition*	2.60 (1.2)	
*Anhedonia*	2.66 (1.30	
CDS	2.92 (3.02)	
HAS	7.15 (5.25)	
Chlorpromazine equivalent [*n* = 134]	790.17 (843.29)	11–4835

*Note.* IQ = intellectual quotient; SAPS = scale for the assessment of positive symptoms; SANS = scale for the assessment of negative symptoms; CDS = Calgary depression scale; HAS = Hamilton anxiety scale.

### Network Examining Symptom Domains

**Figure 1.**
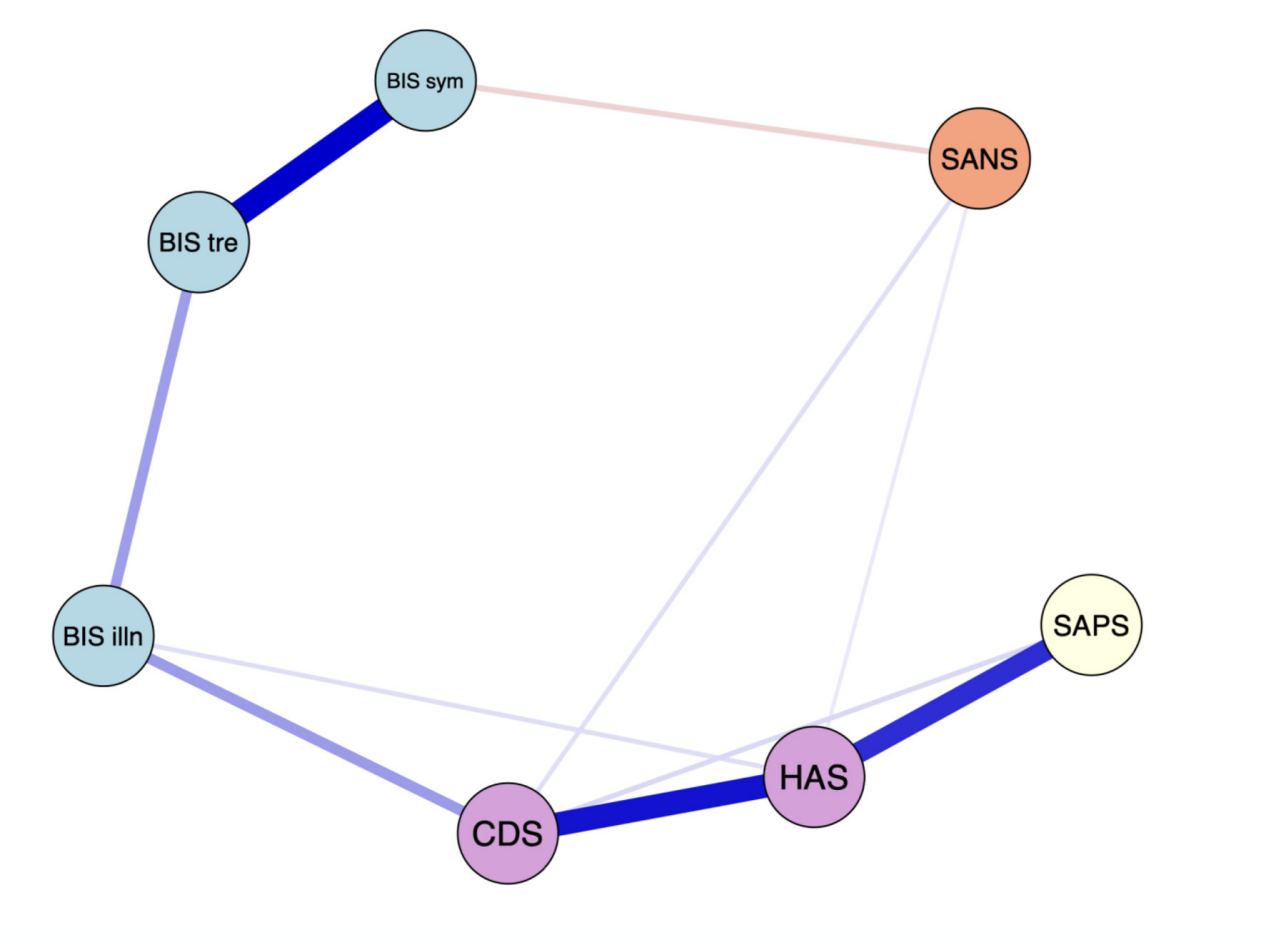
The network graph shows associations estimates between symptom domains and clinical insight dimensions. The edges represent the strength of association between nodes. *Positive associations = blue; negative associations = red; HAS = Hamilton Anxiety Scale; CDS = Calgary Depression Scale; BIS illn = illness awareness; BIS sym = re-labeling of symptoms; BIS tre = awareness of the need for treatment; SAPS = global positive symptoms; SANS = global negative symptoms.

The EBICglasso LASSO network estimation of the total sample is presented in Figure 1. The network has 10 non-zero edges, thus 10 connections among the variables identified as significant. These non-zero edges account for 47.6% of the 21 possible edges, indicating the proportion of significant relationships relative to all potential relationships in the network. The strongest connections, determined by edge weight, were *the re-labelling of symptoms* and *awareness of the need for treatment* (weight = 0.37), depressive symptoms and anxiety symptoms (weight = 0.34), and positive symptoms and anxiety symptoms (weight = 0.31). Regarding insight dimensions, *illness awareness* was most strongly connected to depressive symptoms (weight = 0.14), followed by *awareness of the need for treatment* (weight = 0.14) and anxiety symptoms (weight = 0.03). *The re-labelling of symptoms* showed a small inverse connection with negative symptoms (weight =  -0.07).

**Figure 2.**
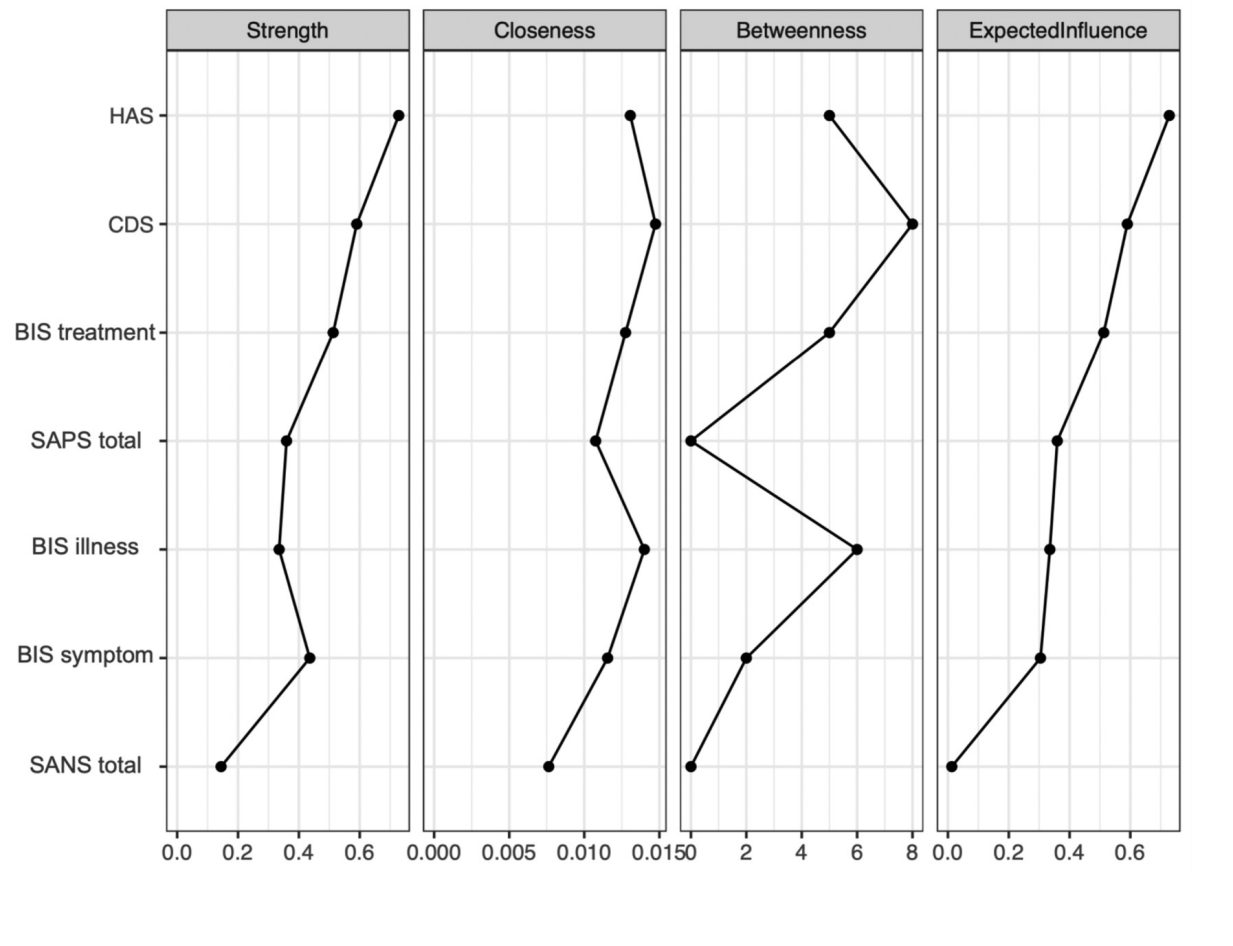
Centrality indices: principal analysis of symptom domains and insight dimensions. HAS = Hamilton Anxiety Scale; CDS = Calgary Depression Scale; BIS treatment = awareness of the need for treatment; SAPS total = global positive symptoms; BIS illness = illness awareness; BIS symptom = re-labeling of symptoms; SANS total = global negative symptoms.

Figure 2 displays the centrality measures. The strength centrality coefficient ranged from 0.01 to 0.72. Anxiety (0.72) and depressive symptoms (0.59) were the variables with the highest strength, indicating they have the most significant influence on the network as a whole. Anxiety symptoms were connected to four variables: depressive symptoms (weight = 0.34), positive symptoms (weight = 0.31), *illness awareness* (weight = 0.05), and negative symptoms (weight = 0.03). Depressive symptoms were also connected to four variables: anxiety symptoms (weight = 0.34), *illness awareness* (weight = 0.14), positive symptoms (weight = 0.05), and negative symptoms (weight = 0.05). *Awareness of the need for treatment* showed the highest strength centrality among insight dimensions (0.51), followed by *illness awareness* (0.38) and then *re-labelling of symptoms* (0.30). Closeness centrality coefficients ranged from 0.007 to 0.014. Depressive symptoms demonstrated the highest value (0.014), followed by *illness awareness* (0.005). Betweenness centrality coefficients ranged from 0 to 16. Depressive symptoms (16) and *illness awareness* (12) demonstrated the greatest betweenness centrality, indicating that the depressive symptoms node serves as a bridge in 16 shortest paths between other nodes, and the illness awareness node serves as a bridge in 12 shortest paths between other nodes.

Depictions of the stability of central indices (CS coefficient) and the accuracy of the estimated network can be found as Supplementary Material. The stability of centrality indices drops steeply for betweenness (CS = 0.03) and closeness (CS = 0.01) but is better for strength (CS = 0.38).

### Exploratory Analysis: Network Examining Symptom Subdomains

**Figure 3.**
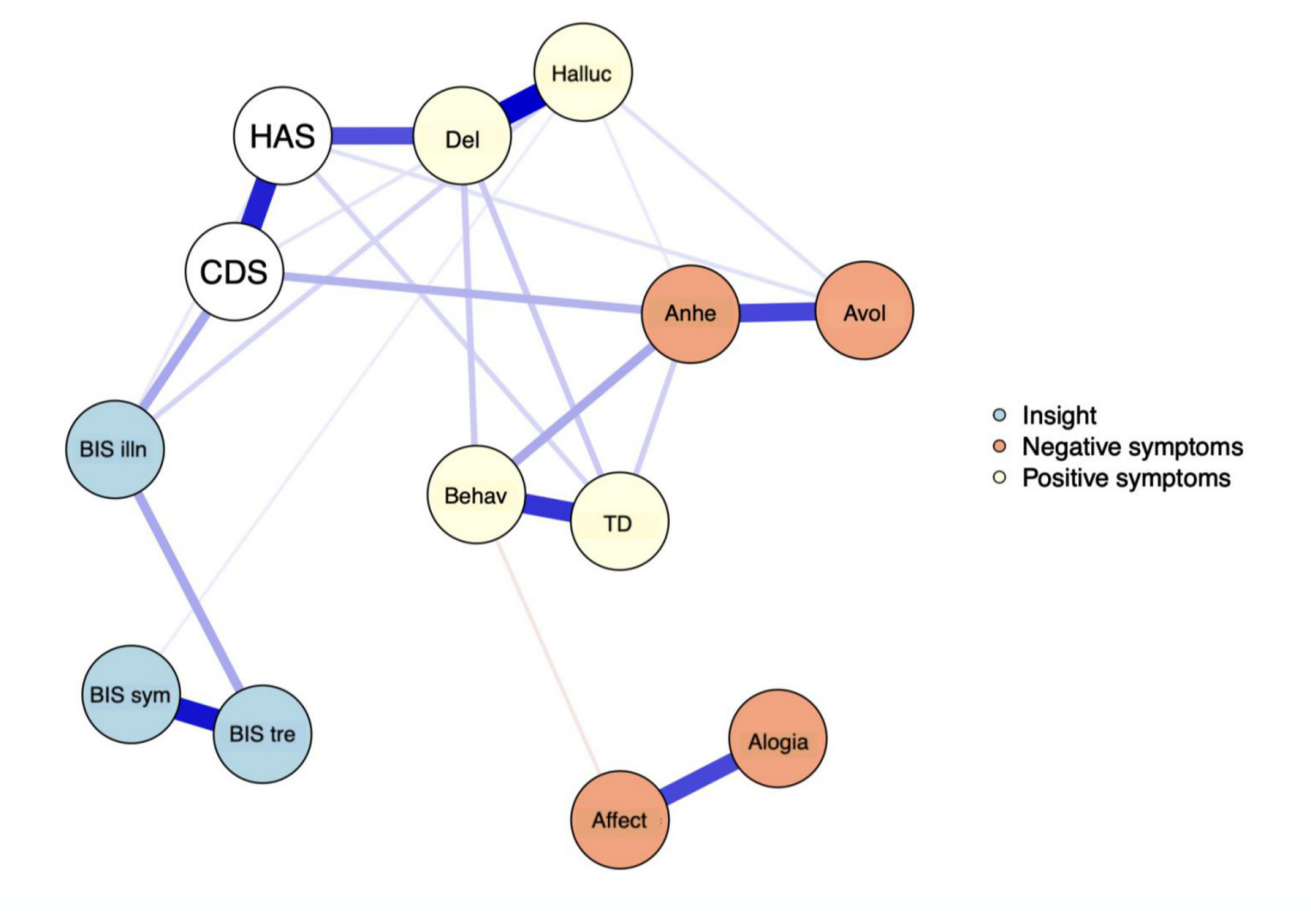
The network graph shows associations estimates between symptom subdomains and clinical insight dimensions. The edges represent the strength of association between nodes. *Positive associations = blue; negative associations = red; HAS = Hamilton Anxiety Scale; CDS = Calgary Depression Scale; BIS illn = illness awareness; BIS sym = re-labeling of symptoms; BIS tre = awareness of the need for treatment; Behav = bizarre behavior; Del = delusions; Halluc = hallucinations; TD = thought disorder; Anhe = anhedonia; Avol = avolition; Affect = affect flattening.

**Figure 4.**
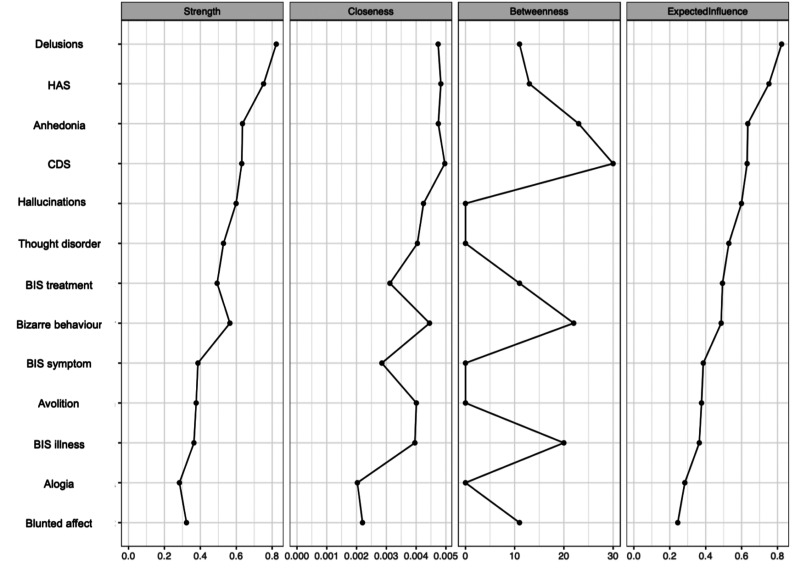
Centrality indices: exploratory analysis of symptom subdomains and insight dimensions. HAS = Hamilton Anxiety Scale; CDS = Calgary Depression Scale; BIS treatment = awareness of the need for treatment; BIS symptom = re-labeling of symptoms; BIS illness = illness awareness.

The EBICglasso LASSO network estimation of the total sample is presented in Figure 3. This network has 23 non-zero edges, thus 23 connections among the variables identified as significant. These non-zero edges account for 29.4% of the 78 possible edges. The strongest connections were hallucinations and delusions (weight = 0.39), *the re-labelling of symptoms* and *awareness of the need for treatment* (weight = 0.36), and bizarre behavior and thought disorder (weight = 0.31). Regarding insight dimensions, *illness awareness* was most strongly connected to depressive symptoms (weight = 0.13), followed by hallucinations (weight = 0.06) and anxiety symptoms (weight = 0.03). *The re-labeling of symptoms* showed a small connection with hallucinations (weight = 0.03). Figure 4 displays the centrality measures. The strength centrality coefficient ranged from 0.33 to 0.87. Delusions (0.87) and anxiety symptoms (0.68) showed the highest strength, thus the most significant influence on the network as a whole. *Awareness of the need for treatment* showed the highest strength centrality (0.48) among dimensions of insight. Depictions of the stability of centrality indices and the accuracy of the estimated network can be found in Supplementary Material. Only strength showed acceptable stability (CS = 0.21).

## Discussion

A network approach was employed to examine the inter-relationships among symptom domains, subdomains, and insight dimensions in individuals with SSD. This study is novel in using a network approach to explore different levels of symptoms and insight dimensions in this population. This method allowed us to visually map the relationships between variables and identify those that are particularly influential in the symptom network, which would not be fully captured through traditional statistical methods. As expected, illness awareness was significantly and positively linked to depressive symptoms. Contrary to our expectations, anxiety and depressive symptoms emerged as the most central, exhibiting the strongest associations with other nodes (as indexed by node strength), suggesting that targeting these symptom domains in treatment could have a broader impact on other symptoms within the clinical profile. Our network stability analysis supports findings from a recent psychometric study, which found node strength more stable than the other two centrality indices.^44^ Theoretical and clinical literature suggests that node strength is particularly relevant and useful for psychopathology networks, as it indicates the likelihood that activating one symptom will trigger the activation of neighboring symptoms.^33^ Therefore, targeting high-strength symptoms might be effective for overall outcomes.

The positive association between illness awareness and depressive symptoms is consistent with a meta-analysis supporting the insight paradox, where increased awareness of illness in patients with schizophrenia can lead to heightened feelings of hopelessness and sadness.^9,14,45–49^ A previous study using the same sample found that awareness of the need for treatment indirectly influenced depressive symptoms through illness engulfment, where an individual's self-concept becomes defined by their illness.^10^ This underscores the importance of addressing mediating factors, like illness engulfment, in interventions, to prevent insight from leading to negative outcomes such as depression. Moreover, our study revealed a positive connection between anxiety and the awareness of the need for treatment. While it is well-documented that anxiety is associated with greater insight in SSD, the relationship between anxiety and specific dimensions of insight remains less explored.^50^ This lack of research may stem from the common practice of conflating anxiety with depression, as these two factors are often linked (as seen in the current study), leading to a combined anxiety/depression score.^9^ However, evidence from this study and others suggests that anxiety and depression may relate to insight differently despite being themselves associated. For instance, patients with schizophrenia and co-occurring anxiety, but not depression, are significantly more likely to seek out medical, psychological, and mental health services than those with no anxiety.^51,52^ Patients with anxious symptoms may be more motivated to seek out treatment, potentially due to the heightened concern inherent in anxiety. Further research is needed to distinguish and better understand the associations between anxiety and depressive symptoms with dimensions of insight, as these nuances may be necessary for improving clinical outcomes.

Depressive and anxiety symptoms demonstrated high strength and betweenness centrality, suggesting that these symptom domains significantly impact other clinical variables within the network, both indirectly and directly. This aligns with previous research demonstrating the influence of depressive symptoms in SSD, including more frequent psychotic episodes, negative symptoms, decreased treatment adherence, increased duration of illness, substance misuse, poor quality of life, and elevated suicide risk.^49,53–57^ Treating depressive and anxiety symptoms may lead to broader improvements across an individual's symptom profile. For example, one study found that reduced depression in schizophrenia patients was linked to improvements in functioning.^53^ Although patients with depressive features typically exhibit better insight, which has been associated with improved treatment adherence, numerous studies have reported decreased treatment adherence in this population.^53,58–61^ It is possible that the observed nonadherence among depressive patients may exacerbate other symptoms, creating a vicious cycle that intensifies their overall clinical condition. This also challenges the notion that increased insight necessarily leads to better treatment adherence and suggests that illness awareness alone may not suffice to achieve positive clinical outcomes.^53,62^ Understanding insight as a multidimensional construct can provide a clearer picture of how its various dimensions influence different clinical outcomes, such as treatment adherence.

Awareness of the need for treatment emerged as the most influential dimension of insight, thus significantly impacting other symptoms in the network. Despite no direct association with depressive symptoms, some research has shown that patients with SSD may lack awareness in this particular dimension when concurrently experiencing depressive symptoms. For instance, one study found that while awareness of the disorder was better in patients with psychosis and comorbid depression, the ability to recognize the need for treatment was similar across diagnostic groups.^63^ Future research could benefit from exploring how depressive and anxiety symptoms, and insight dimensions relate to treatment nonadherence in SSD to better understand the relationship between these variables. This could, in turn, promote rehabilitation, by improving patient-clinician communication and reducing the risks of relapse and hospitalization.

In conjunction with the number of variables, a larger sample size may have more effectively detected significant patterns, relationships, or differences within the network.^64^ Although the strength centrality measure demonstrated sufficient reliability, the stability analysis revealed that only a small portion of the sample could be excluded while maintaining the study's findings related to closeness and betweenness. With a larger sample, the centrality measure's reliability would improve, enhancing the interpretability of these measures. Additionally, the Birchwood IS has limitations, including potential bias from self-reporting and its failure to assess awareness of the social consequences of one's illness. The wide range of illness duration introduces heterogeneity to our sample, which could impact the results, given that insight has been shown to change over time.^65^ Specifically, the relationships between symptoms and insight dimensions vary based on illness duration. Future research should use a longitudinal approach to measure insight over time and explore how these fluctuations may relate to changes in symptom severity. Including other relevant variables in a network, such as treatment adherence, medication effects, illness severity or cognitive areas affected in psychosis, could provide a deeper understanding of how clinical variables influence and are influenced by one another. Overall, the current study's limitations highlight the need for a more extensive investigation with a larger sample size to enhance the interpretability of centrality indices and allow for the possibility of including additional variables for a more comprehensive understanding of symptom networks in SSD.

## Conclusion

In this study, we employed a network approach to examine the inter-relationships among symptom domains, subdomains, and insight dimensions in individuals with SSD. Our findings highlight the centrality of depressive and anxiety symptoms within the network, suggesting their significant influence on other clinical variables. Targeting depression and anxiety in treatment with SSD may produce a positive impact on other symptoms. The association between illness awareness and depressive symptoms supports previous research demonstrating that patients with better insight are more likely to also experience depressive symptoms, potentially leading to worse clinical outcomes. Additionally, the positive association between anxiety and the awareness of the need for treatment points to a complex interaction where anxiety may drive treatment-seeking behaviors, yet its coexistence with depression could complicate adherence and overall prognosis. Future research should further explore these relationships within a larger sample to inform targeted interventions aimed at improving treatment adherence and reducing relapse in SSD.

## Supplemental Material

sj-docx-1-cpa-10.1177_07067437251329074 - Supplemental material for Mapping Insight Dimensions and Symptom Dynamics in Schizophrenia: A Data-Driven Network Approach: Cartographie des dimensions d’insight et de la dynamique symptomatique dans la schizophrénie: une approche par réseau fondée sur les données Supplemental material, sj-docx-1-cpa-10.1177_07067437251329074 for Mapping Insight Dimensions and Symptom Dynamics in Schizophrenia: A Data-Driven Network Approach: Cartographie des dimensions d’insight et de la dynamique symptomatique dans la schizophrénie: une approche par réseau fondée sur les données by Jesse Rae, Katie M. Lavigne, Geneviève Sauvé, Martin Lepage and Delphine Raucher-Chéné in The Canadian Journal of Psychiatry
