# Supplementary material for: Mapping Insight Dimensions and Symptom Dynamics in Schizophrenia: A Data-Driven Network Approach: Cartographie des dimensions d’insight et de la dynamique symptomatique dans la schizophrénie: une approche par réseau fondée sur les données
Source: Can J Psychiatry. 2025 Mar 21;70(4):301–11. doi: 10.1177/07067437251329074 (PMC11930468; doi:10.1177/07067437251329074)
Supplement: sj-docx-1-cpa-10.1177_07067437251329074 - Supplemental material for Mapping Insight Dimensions and Symptom Dynamics in Schizophrenia: A Data-Driven Network Approach: Cartographie des dimensions d’insight et de la dynamique symptomatique dans la schizophrénie: une approche par réseau fondée sur les d [file sj-docx-1-cpa-10.1177_07067437251329074.docx]

**Supplementary material**

**Methods**

Both estimations were performed using the qgraph and bootnet packages, representing a Gaussian Graphical Model (GGM), where nodes represent variables, and edges represent partial correlation coefficients, after controlling for other connections.^1, 65^

A weighted undirected network, which indicates mutual relationships between nodes and is typically used for cross-sectional data, was produced using the qgraph package. ^1, 65^ The Fruchterman−Reingold algorithm was employed to organize the plot, placing more strongly connected nodes closer together and nodes with higher centrality indices nearer to the center of the graph. ^2^ Least Absolute Shrinkage and Selection Operator (LASSO) is a method for estimating sparse linear regression models used in network estimation when dealing with high-dimensional data. The extended Bayesian information criterion graphical least absolute shrinkage (EBICglasso) procedure, a specific variant of the LASSO method, was applied to control for false positive edges, resulting in a sparser network where the absence of an edge indicated conditional independence. ^3^

1. Epskamp S, Cramer AOJ, Waldorp LJ, Schmittmann VD, Borsboom D. qgraph: Network Visualizations of Relationships in Psychometric Data. *Journal of Statistical Software*. 2012;48(4):1–18.
2. Fruchterman TM, Reingold EM. Graph drawing by force‐directed placement. *Software: Practice and Experience*. 1991;21(11):1129-1164.
3. Foygel R, Drton M. Extended Bayesian information criteria for Gaussian graphical models. *Advances in Neural Information Processing Systems*. 2010;23.

Stability of centrality indices by case dropping subset bootstrap: principal analysis of symptom domains and insight dimensions.


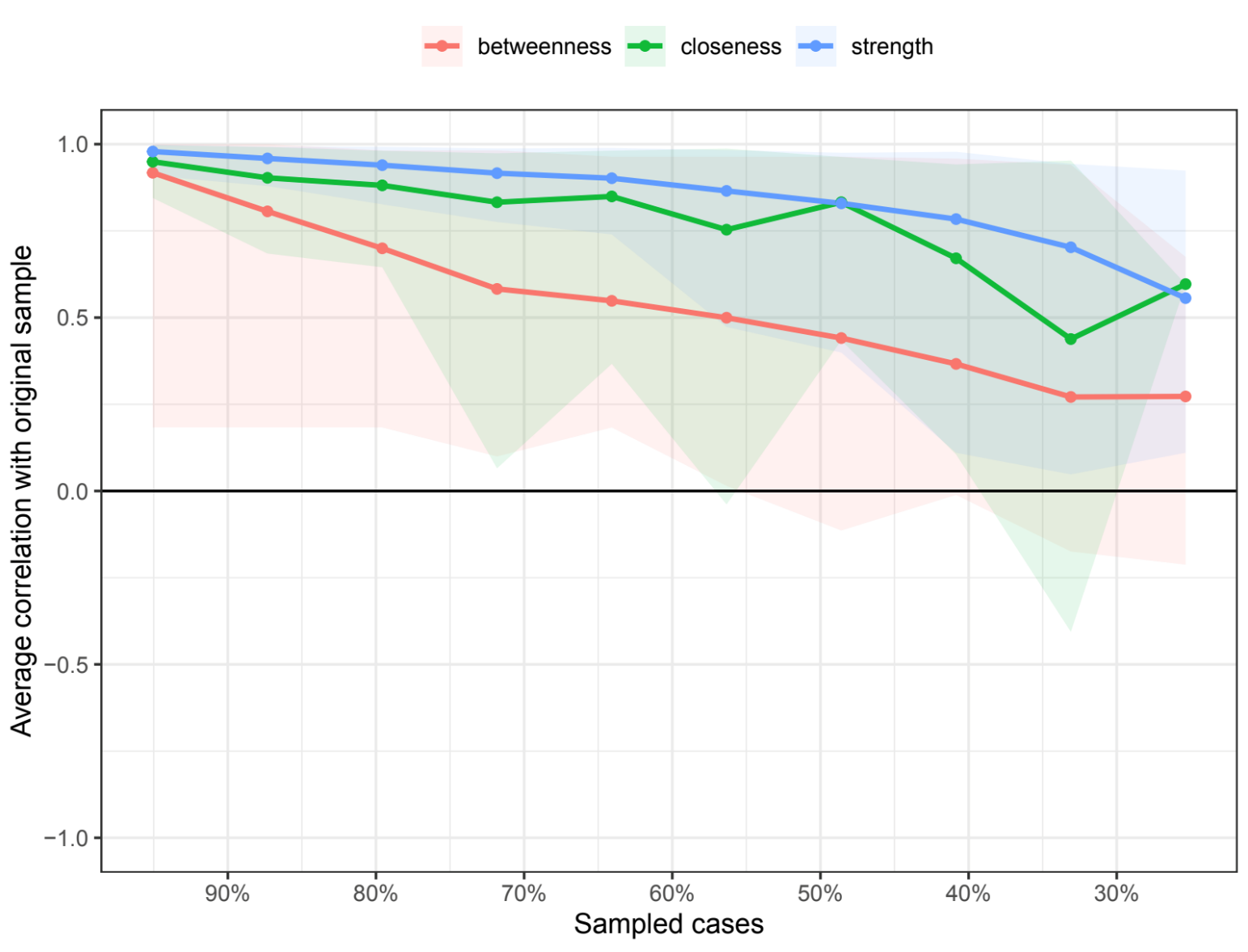


*Note.* The x-axis represents the percentage of cases retained from the original sample at each step, while the y-axis represents the average correlations between the centrality indices of the original network and those of networks re-estimated after progressively removing more cases. Each line corresponds to the correlations for betweenness, closeness, and strength, with the shaded areas indicating the 95% confidence intervals.

Stability of edge-weights: principal analysis of symptom domains and insight dimensions.


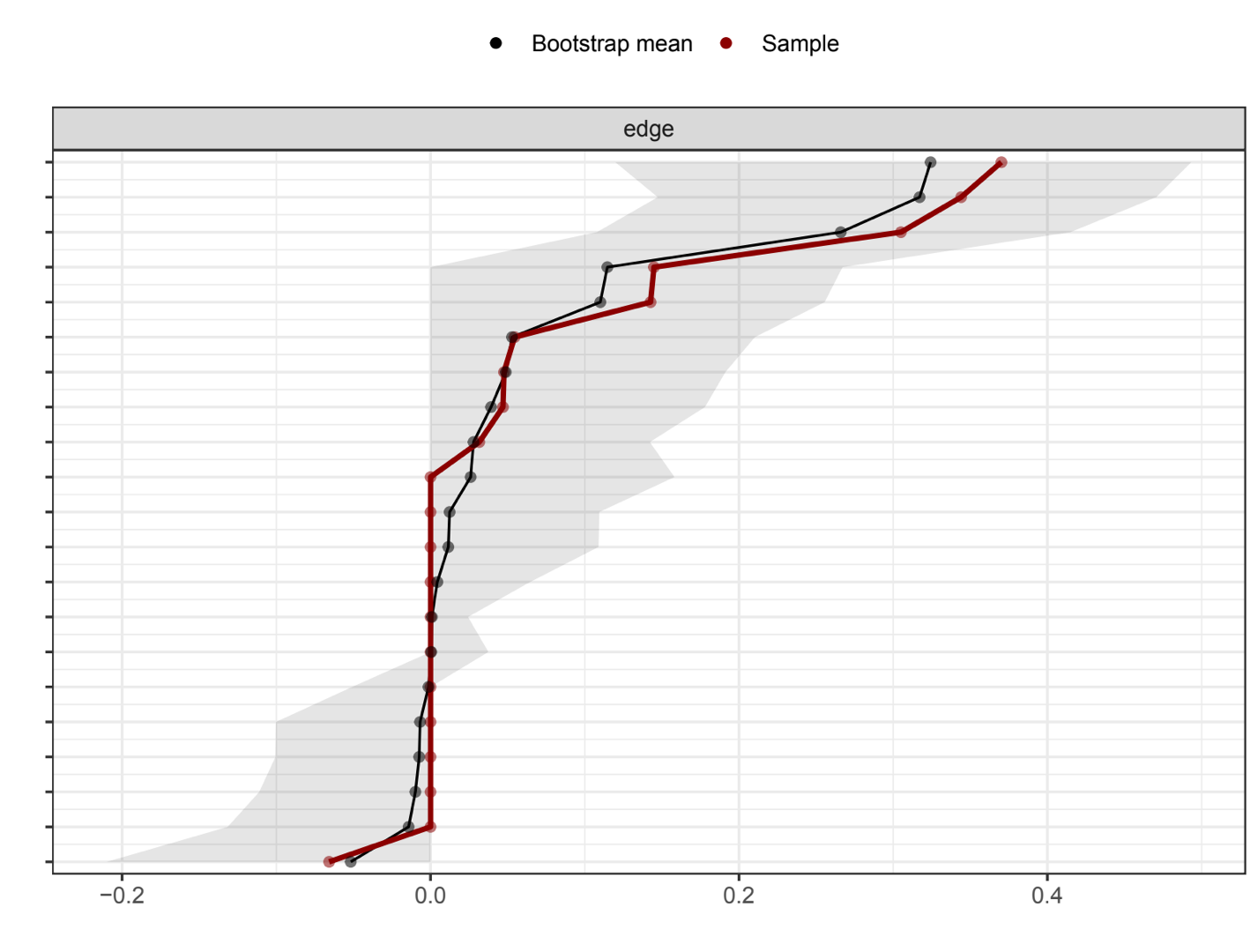


*Note.* The red line represents the sample values, while the grey area shows the bootstrapped 95% confidence intervals. Each horizontal line on the y-axis corresponds to one edge of the network, arranged from the highest to the lowest edge weight. The y-axis labels have been removed to reduce clutter.
